# Concordance rate between copy number variants detected using either high- or medium-density single nucleotide polymorphism genotype panels and the potential of imputing copy number variants from flanking high density single nucleotide polymorphism haplotypes in cattle

**DOI:** 10.1186/s12864-020-6627-8

**Published:** 2020-03-04

**Authors:** Pierce Rafter, Isobel Claire Gormley, Andrew C. Parnell, John Francis Kearney, Donagh P. Berry

**Affiliations:** 1 Animal & Grassland Research and Innovation Centre, Teagasc, Moorepark, Fermoy, Co. Cork, Ireland; 2 UCD School of Mathematics and Statistics, University College Dublin, Belfield, Dublin 4, Ireland; 3 Hamilton Institute Maynooth University, Maynooth, Kildare, Ireland; 4 Irish Cattle Breeding Federation, Highfield House, Shinagh, Bandon, Co., Cork, Ireland

**Keywords:** CNV, Bovine, PennCNV, QuantiSNP, Beagle, FImpute, SNP, Imputation

## Abstract

**Background:**

The trading of individual animal genotype information often involves only the exchange of the called genotypes and not necessarily the additional information required to effectively call structural variants. The main aim here was to determine if it is possible to impute copy number variants (CNVs) using the flanking single nucleotide polymorphism (SNP) haplotype structure in cattle. While this objective was achieved using high-density genotype panels (i.e., 713,162 SNPs), a secondary objective investigated the concordance of CNVs called with this high-density genotype panel compared to CNVs called from a medium-density panel (i.e., 45,677 SNPs in the present study). This is the first study to compare CNVs called from high-density and medium-density SNP genotypes from the same animals. High (and medium-density) genotypes were available on 991 Holstein-Friesian, 1015 Charolais, and 1394 Limousin bulls. The concordance between CNVs called from the medium-density and high-density genotypes were calculated separately for each animal. A subset of CNVs which were called from the high-density genotypes was selected for imputation. Imputation was carried out separately for each breed using a set of high-density SNPs flanking the midpoint of each CNV. A CNV was deemed to be imputed correctly when the called copy number matched the imputed copy number.

**Results:**

For 97.0% of CNVs called from the high-density genotypes, the corresponding genomic position on the medium-density of the animal did not contain a called CNV. The average accuracy of imputation for CNV deletions was 0.281, with a standard deviation of 0.286. The average accuracy of imputation of the CNV normal state, i.e. the absence of a CNV, was 0.982 with a standard deviation of 0.022. Two CNV duplications were imputed in the Charolais, a single CNV duplication in the Limousins, and a single CNV duplication in the Holstein-Friesians; in all cases the CNV duplications were incorrectly imputed.

**Conclusion:**

The vast majority of CNVs called from the high-density genotypes were not detected using the medium-density genotypes. Furthermore, CNVs cannot be accurately predicted from flanking SNP haplotypes, at least based on the imputation algorithms routinely used in cattle, and using the SNPs currently available on the high-density genotype panel.

## Background

A copy number variant (CNV) is a form of genetic variation that arises from a deletion or duplication of a stretch of DNA [1]. By convention, CNVs typically have a minimum length of 1 kb; deletions or duplications that are shorter are usually considered to be indels [2]. Copy number variants are a common feature of the bovine genome, with the average number of CNVs per individual, identified from high-density genotype data, ranging from 18 to 51 [3–5]. In cattle, there are reported associations between CNVs and milk production [6], meat tenderness [7], and health traits [8].

Several software suites exist to call CNVs from single nucleotide polymorphism (SNP) data now routinely generated from what are commonly called SNP-chips or beadchips [9]. PennCNV [10] and QuantiSNP [11] are two such software suites and both algorithms use the Log R Ratio (LRR) and B allele frequency (BAF) values of SNPs to call CNVs. Where the LRR or BAF values are not available, CNVs cannot be identified. Such situations may exist where genotypes have been exchanged among parties [12], where only the called genotype was exchanged, but also in situations where the LRR and BAF were historically not stored. If CNVs can be accurately imputed from SNP haplotypes flanking the CNV, then CNVs could be called from SNP data that lacks LRR or BAF values.

Microsatellites, which are structurally similar to CNVs, have previously been imputed from flanking SNPs genotyped using a high-density SNP genotype panel in more than 8000 cattle; the median imputation accuracy was 72%, but the accuracy of imputation for some microsatellites was up to 100% [13]. The objective of the present study was to quantify the accuracy of imputing CNVs detected using CNV calling algorithms from the haplotypes of flanking high density SNPs in cattle. Given the greater usage of medium-density genotypes (c.a. 50,000 SNPs) relative to high-density genotypes (c.a. 777,000 SNPs) in cattle, of particular interest in the present study was also the concordance between CNVs called from high-density SNP platforms and CNVs called from medium-density SNP platforms.

## Results

### Comparison of CNVs called from the high-density and medium-density genotypes

PennCNV called a total of 10,971 CNVs from the medium-density genotypes and a total of 159,046 CNVs from the high-density genotypes across all three breeds; this included both novel CNVs and CNVs called in more than one individual. The median number of CNVs per animal called from the medium-density and high-density genotypes were 2 and 27, respectively. Summary statistics for the CNVs called from the high-density genotypes that overlapped with CNVs called from the medium-density genotypes are presented in Table 1. For all three breeds, CNVs called from the high-density genotype panel whose genomic position overlapped with a CNV called from the medium-density genotype were, on average, longer than CNVs detected on the high-density genotypes whose genomic position did not overlap with any CNVs detected on the medium-density genotype (*p* < 0.05). Irrespective of breed, CNVs called from high-density genotypes whose genomic position overlapped with CNVs called from the medium-density genotypes occurred less frequently in the population than the CNVs that had no overlap (*p* < 0.05). For 97.0% of the CNVs called from the high-density genotypes, a CNV was not detected in the same genomic region of the same animal using the medium-density genotype. For 87.4% of the CNVs called from the high-density genotypes, the same genomic region on the medium-density genotype had less than 3 SNPs; therefore a CNV could never be called in those genomic regions using the medium-density genotype panel because PennCNV requires a minimum of 3 SNPs to be called.

**Table 1:** The first quartile, median, and third quartile for the genomic length, and the number of SNPs per CNV for the CNVs called from the high-density genotypes. The CNVs called from the high-density genotypes are grouped separately based on the degree of overlap of the genomic position of the CNVs called from the high and medium density genotypes. Direct overlap is where is the genomic position of both CNVs were the same, partial overlap is where the genomic positions partially overlapped, and no overlap is where the genomic positions of the CNVs did not overlap

	Count	Q1 length (kb)	Median length (kb)	Q3 length (kb)	Q1 number of SNPs	Median number of SNPs	Q3 number of SNPs
Direct overlap	19	77.3	115.2	165.8	13	22	39
Partial overlap	4828	61.4	139.8	279.4	18	41	80
No overlap	154,199	14.8	36.1	79.8	5	11	23

### Imputation

**Fig. 1:**
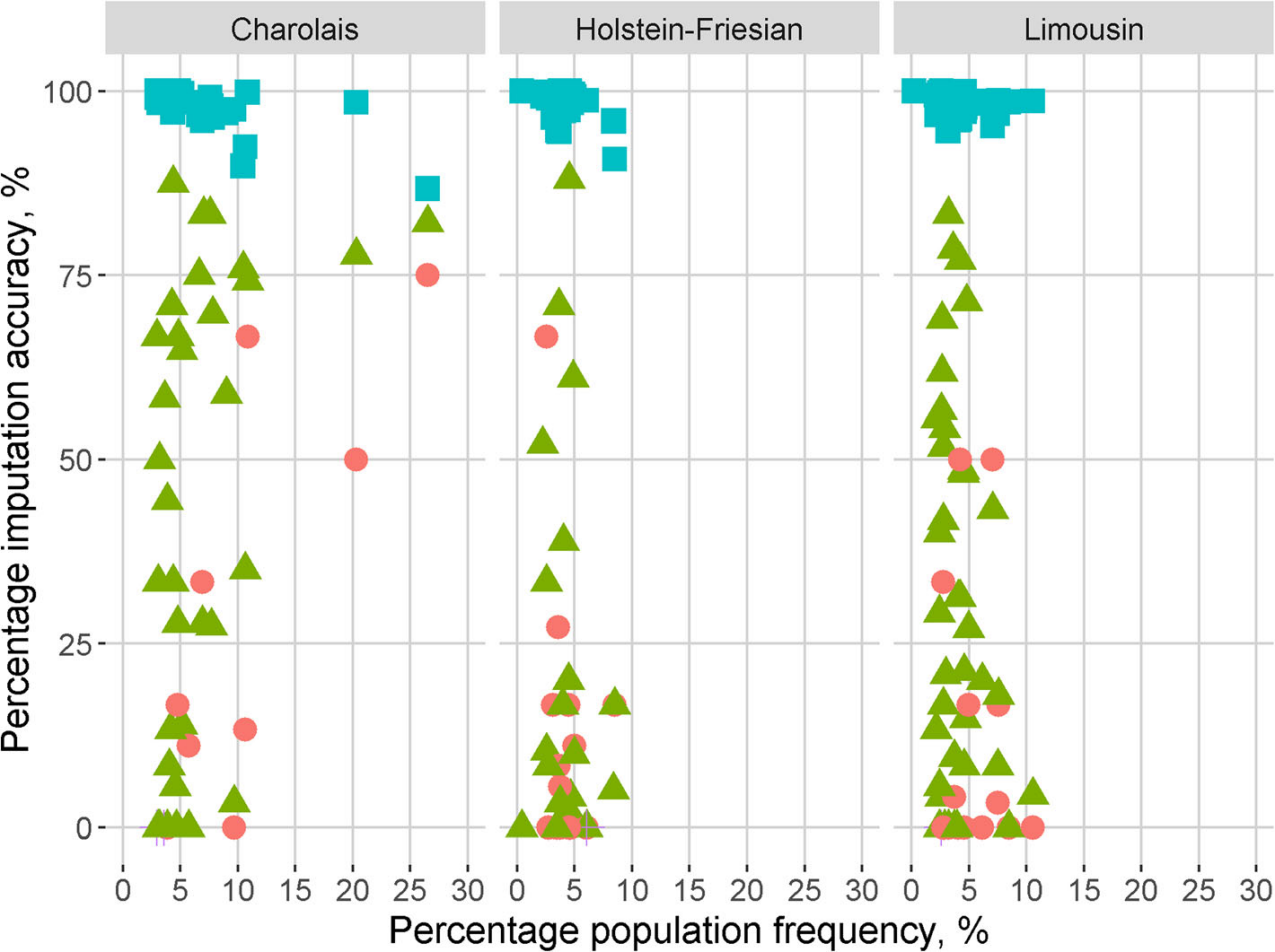
Scatter plot of the percentage imputation accuracy against the percentage population frequency of each CNV. A CNV was deemed to be correctly imputed when the called copy number matched the imputed copy number. The red circles represent double deletions (*n* = 9 in Charolais, 15 in Limousin and Holstein-Friesian), green triangles represent single deletions (*n* = 34 in Charolais, *n* = 38 in Limousin, and 22 in Holstein-Friesian), blue squares represent normal state (*n* = 36 in Charolais, 40 in Limousin, and 24 in Holstein-Friesian), double duplications are represented by a purple cross (*n* = 2 in Charolais, *n* = 1 in Limousin and Holstein-Friesian)

**Fig. 2:**
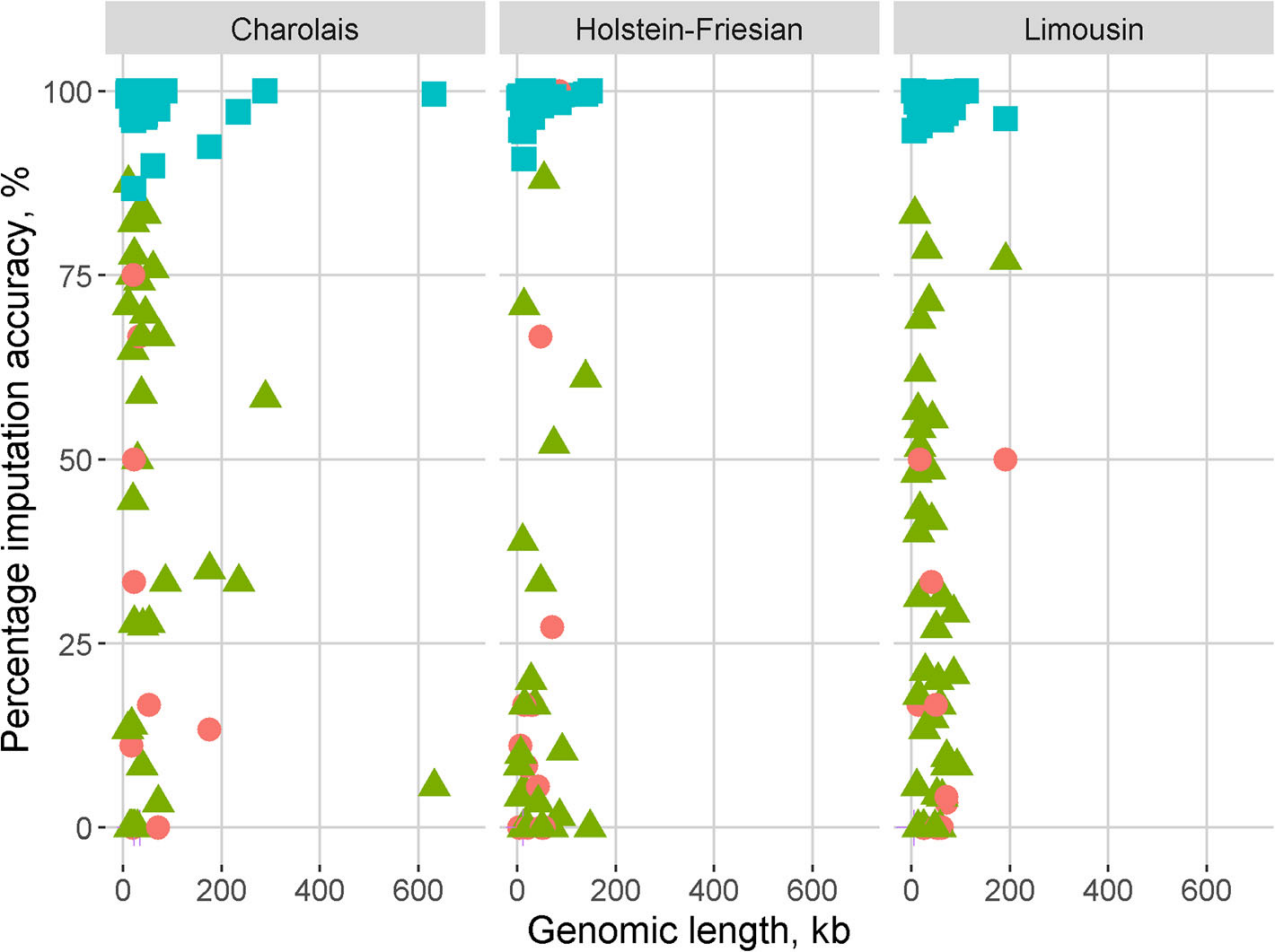
Scatter plot of percentage imputation accuracy against genomic length of CNVs. A CNV was deemed to be correctly imputed when the called copy number matched the imputed copy number. The red circles represent double deletions (n = 9 in Charolais, 15 in Limousin and Holstein-Friesian), green triangles represent single deletions (n = 34 in Charolais, n = 38 in Limousin, and 22 in Holstein-Friesian), blue squares represent normal state (n = 36 in Charolais, 40 in Limousin, and 24 in Holstein-Friesian), double duplications are represented by a purple cross (n = 2 in Charolais, n = 1 in Limousin and Holstein-Friesian)

The accuracy of imputing CNVs was similar for both FImpute and Beagle, and thus, only the results relating to imputation using FImpute are presented; results relating to imputation with Beagle are presented in the additional files. The normal state (i.e. the absence of a CNV) was imputed with a greater accuracy than deletions or duplications (*p* < 0.05). The summary statistics regarding the accuracy of imputation for deletions and the absence of a CNV are presented in Table 2. Two duplications were imputed in the Charolais, one in the Limousins, and one in the Holstein-Friesians; in all cases, the imputed copy number did not match the called copy number. There was no difference in the accuracy of imputation between the breeds, except for single deletions which were more accurately imputed in Charolais than in Holstein-Friesians (*p* < 0.05). Irrespective of breed, the accuracy of imputing the CNV genotypes was not influenced by the number of flanking SNPs used in the imputation process. The relationship between the accuracy of imputation and the population frequency of the CNV, and the relationship between the accuracy of imputation and the genomic length of the CNV is in Figs. 1 and 2, respectively; neither of the correlations differed from zero for any of the three breeds. In Holstein-Friesians, CNVs which were accurately imputed had, on average, a higher Bayes factor than CNVs inaccurately imputed (*p* < 0.05), whereas in the Limousins the opposite was true (p < 0.05). In the Charolais, and all three breeds combined, there was no difference in the Bayes factor between CNVs where the called and imputed copy number matched versus CNVs where the imputed and called copy number did not match.

**Table 2:** The first quartile, median, and third quartile of the accuracy of imputation of CNVs grouped by called copy number and breed. The number of CNVs in each group is also given. Summary statistics for duplications (*n* = 4) were not included because for each duplication the imputed copy number did not match the called copy number

	Breed	First quartile	Median	Third quartile	Number of CNVs
Double deletions	Charolais	0.110	0.167	0.500	9
	Limousin	0.000	0.000	0.167	15
	Holstein-Friesian	0.000	0.083	0.167	15
Single deletions	Charolais	0.096	0.397	0.705	34
	Limousin	0.083	0.241	0.509	38
	Holstein-Friesian	0.004	0.092	0.300	22
Normal	Charolais	0.974	.991	0.997	36
	Limousin	0.978	0.985	0.994	40
	Holstein-Friesian	0.974	0.987	0.994	24

In addition to the imputation accuracy, the adjusted Rand Index was calculated separately for each breed to quantify the agreement between the called copy number and the imputed copy number of a CNV. The adjusted Rand index was 0.524 for Charolais, 0.361 for the Limousins, and 0.285 for the Holsteins-Friesians meaning there was more similarity between the called copy number and the imputed copy number of the CNVs than was expected by chance, albeit not a very strong agreement, given the maximum value the adjusted Rand index can take is 1.

In the present study, most CNVs were imputed with low accuracy; however, there were some CNVs which had an imputation accuracy of at least 85% within breed. The CNVs with an accuracy of at least 85% are presented in Table 3.

**Table 3:** The location and population frequency of CNVs with an accuracy of at least 85% within at least one of the three breeds. The population frequency is the number of times the CNV was present in the total population, i.e. the reference and validation population. The accuracy, given as a percentage, is the number of times the CNV was accurately imputed divided by the number of times that CNV was called in the validation population. Where an accuracy of NA is reported, imputation was not undertaken for that CNV in that breed

CNV genomic location	Limousin		Charolais		Holstein-Friesian	
	Population frequency	Accuracy, %	Population frequency	Accuracy, %	Population frequency	Accuracy, %
12:72,174,261–72,259,734	40	20.8	29	NA	47	100
7:10,216,191–10,270,468	0	NA	0	NA	45	88.1
5:41517287–41,528,650	2	NA	44	87.5	0	NA

## Discussion

Associations between CNVs and phenotypic performance have been documented in a whole multitude of species including dairy cattle [6], beef cattle [7, 8], chickens [14], dogs [15], pigs [16] and humans [17–19]; thus CNVs are likely to contribute to some of the underlying genetic variability. The ability to estimate the genomic or phenotypic merit of individuals based on CNVs requires knowledge of the CNV genotypes of those animals. Specialized calling algorithms are generally used to detect CNVs from SNP genotype data [10, 11], with most studies opting to use either PennCNV [4, 20, 21] or QuantiSNP [21–23]; these were the two calling algorithms used in the present study. The density of genotype panels used in CNV-based studies in cattle varies from circa 50,000 SNPs [24–26] to over 700,000 SNPs [21, 27, 28]. Little, however, is known of the ability of circa 50,000 SNP panels to detect CNVs identified from higher density SNP panels; this is particularly important given the greater usage of medium-density (i.e. circa 50,000 SNPs) genotype panels in domesticated species.

### Comparison of CNVs called from the high-density and medium-density genotypes

The present study is the first such in cattle to directly compare CNVs called from medium-density and high-density genotypes in the same animals. PennCNV requires a minimum of 3 SNPs to call a CNV; for 84.7% of CNVs called from the high-density genotypes, the same genomic region of the CNV on the medium-density genotype panel had less than three SNPs. Therefore those CNVs could never have been called using the medium-density genotypes. Even though no study, to date, has compared the concordance of CNVs called from high-density genotypes versus medium-density genotypes in the same cattle, the trend observed in the literature is that more CNVs are called from high-density genotypes than medium-density genotypes. In cattle, typically between 18 and 51 CNVs are called per animal from high-density genotypes (c.a. 700,000 SNPs) [3–5], whereas other cattle studies using medium-density genotypes (c.a. 50,000 SNPs) have reported between 1 and 7 CNVs per animal [25, 26], which is consistent with the results of the present study.

The CNVs called from the high-density genotypes whose genomic position overlapped with CNVs called from the medium-density genotype panel had a lower population frequency than CNVs with no overlap between panels. This is in line with expectations because longer CNVs were more frequently overlapped and it has previously been shown that longer CNVs tend to have a lower population frequency [21]. In a study somewhat similar to the present study, Purfield et al. [29] compared genomic features, known as runs of homozygosity, called from high-density but also from masked genotypes on the same cattle to mimic a medium-density panel; Purfield et al. [29] reported that runs of homozygosity were more frequently identified from the higher-density genotypes than from medium-density genotypes. Furthermore, there was a positive relationship between the length of the run of homozygosity identified from the high-density genotypes and the probability of overlap with a run of homozygosity identified from the medium-density genotype in the same animal [29]. This pattern of overlap is analogous to the pattern of overlap observed in the present study for CNVs called from the medium-density and high-density genotypes.

The median number of CNVs called per animal from the medium-density genotype in the present study was 2, but it was 27 for the high-density genotypes; given that the false positive rate of CNVs called from PennCNV and QuantiSNP is reported to be 1–2% [10, 11, 22] it suggests that most of the CNVs called from the high-density genotype panel are in fact true CNVs. Therefore, many CNVs probably cannot be detected using the medium-density genotype panel. Moreover, it may be hypothesized that the number of CNVs detected with the high-density genotypes is only a fraction of those that truly exist and could be detected with ultra-high-density genotypes (i.e., sequence). In cattle, many more CNVs are called using whole genome sequence than from high-density genotype data; Kommadath et al. [30] reported that the average number of CNVs called from whole genome sequence data is 304 CNVs per animal in cattle. By comparison for high-density genotype data, the average number of CNVs per animal is reported to be between 18 and 51, as mentioned previously. It may be the case that many of the additional CNVs called from whole genome sequence are true CNVs that cannot or are unlikely to be called from high-density SNP data. A possible reason for this is that short CNVs may be present in genomic regions in-between genotyped SNPs on panels, or do not encompass the required minimum number of genotyped SNPs to be called by a CNV calling algorithm; in the case of PennCNV, three SNPs are required to call a CNV.

Another possible factor that might limit the ability of high-density SNP genotype data to detect CNVs is bias in SNP selection for commercially available SNP genotype panels. One of the selection criteria for including SNPs on a genotype panel is high genotyping accuracy [31]. The SNPs which do not adhere to expected Mendelian inheritance patterns, and the SNPs that have poor genotyping clustering scores tend to be considered genotyping errors, and as such, tend not to be included in genotype panels. While genotyping error can cause Mendelian inconsistencies and poor genotype clustering, both can also be caused by the presence of a CNV or indel [32]. Therefore, genomic regions that are frequently subject to copy number variation may be poorly represented by SNPs on genotype panels.

### Imputation

Imputation of CNVs from flanking SNPs genotypes has not previously been attempted in cattle although it has been studied in humans [33]. Handsaker et al. [33] used Beagle V4.0 to impute CNV duplications called from whole genome sequence in 849 people sequenced as part of the 1000 Genomes Project; the CNVs in that study were called using the Genome STRiP algorithm [34]. Handsaker et al. [33] reported that the correlation between the actual copy number and the imputed copy number of a CNV was uniformly distributed between 0 and 100% with an average accuracy of approximately 50%. Similarly, in the present study there was a wide range in CNV imputation accuracy within each of three breeds (Fig. 1.).

Su et al. [35] developed the polyHap 2.0 software package to impute the copy number of SNPs from genotype data. Their dataset consisted of CNVs called from bespoke SNP genotypes (i.e., 244,000 SNPs) of 48 French human males with the ADM2 CNV calling algorithm, and CNVs called from Illumina Hap 370 genotypes of 695 Finnish human males using PennCNV and QuantiSNP. Su et al. [35] deemed the copy number of a SNP to be correctly imputed when the called copy number matched the imputed copy number. They reported an imputation accuracy of between 91 and 100% in the 48 French human males, and an imputation accuracy of between 92 and 97% in the 695 Finnish human males. In the present study, as well as in the study of Handsaker et al. [33], the validation populations contained only the genotype data of the flanking SNPs/nucleotides; in contrast, Su et al. [35] imputed to a validation population in which the copy number and genotypes of the flanking SNPs was actually known. Given that Su et al. [35] imputed to a validation population in which the copy number state of the flanking SNPs was known, it is expected that imputation would be more accurate than if the copy number of the SNPs in the validation population was not known. This is because a CNV is a continuous stretch of DNA that displays a gain or loss in copy number and therefore the copy number of an individual SNP can often be inferred from the copy number of its flanking SNPs.

The average accuracy of imputation for the deletion CNVs in the present study was 28.6%, meaning that across all animals with a called deletion CNV, the called copy number matched the imputed copy number in only 28.6% of cases. For all 4 duplication CNVs examined, the imputed copy number never matched the called copy number. By comparison the average accuracy of imputation for SNPs in cattle is reported to be > 90% [36–38], while the average accuracy of imputation for microsatellites was reported to be 72% [13]. The low imputation accuracy of CNVs relative to both SNPs and microsatellites could be due to several reasons. Firstly, in the present study, the imputed genotype of CNVs was compared to the called genotype of CNVs; therefore low accuracy could be a result of inaccurate CNV calling or inaccurate CNV imputation. In this study, to be more confident in the CNVs called, only CNVs which were called by both PennCNV and QuantiSNP were examined. Furthermore, across all three breeds, the Bayes factor of CNVs was not different between the CNVs whose called copy number matched the imputed copy number and the CNVs whose called and imputed copy number did not match. Taken together, this indicates that false positive CNVs in the reference and validation populations probably did not impact much the accuracy of imputation. For the present study, false negative CNV calls could, in part, be accounted for by using pedigree information. Using pedigree information, opposing homozygous CNVs present in sire-progeny pairs can be identified, and opposing homozygous CNVs may have arisen from false negative CNV calls. For both FImpute and Beagle, imputation was carried out using pedigree information.

Another possible reason for the low accuracy of imputation could also be due ascertainment bias in the SNP selection criteria for SNP genotype panels. The SNPs used in SNP imputation studies [36–38] are SNPs on commercially available genotyping panels; one of the selection criteria for SNPs to be included on a genotype panel is high minor allele frequency (MAF) [31, 38, 39]. The microsatellites used in the McClure et al. [13] study were microsatellites that had been commonly used for parentage verification in cattle. Similar to the SNPs on the commercially available genotype panels, these microsatellites also had high MAF in the cattle population [13]; in contrast, CNVs tend to be rare [7, 20, 21]. The difference in the MAF between CNVs and the SNPs used to impute those CNVs may therefore contribute to the low imputation accuracy of CNVs. This is because imputation relies on linkage disequilibrium between the known (i.e., genotyped) variants and the missing variants; common variants cannot be in complete linkage disequilibrium with a rarer variant because there has to be cases where the common variant is present and the rare variant is absence. Therefore, the low accuracy of imputation of CNVs in the present study could be because the SNPs flanking the CNV had a higher frequency in the population than the CNV. Successful imputation of CNVs from SNP genotype data may require the use of SNPs which have a MAF similar to the MAF of the CNVs to be imputed.

## Conclusions

In this study CNVs could not be accurately detected using SNP haplotype data available on the BovineHD SNP chip. Current CNV detection algorithms rely on the LRR and BAF values to detect CNVs; where genotype data are exchanged between parties, the LRR and BAF will have to be included with the genotype data to facilitate CNV detection. Where it is known that a CNV is associated with, or contributes to a phenotype, that region of the genome should be more densely populated with SNPs on a SNP genotype panel enabling improved accuracy in the identification of CNVs associated with production in cattle. Overall, this could contribute to improved genomic and phenotypic predictions.

## Methods

### Genotype data

BovineHD BeadChip (Illumina Inc., San Diego, CA) genotypes, which included LRR and BAF information for all SNPs, were available on 1015 Charolais, 991 Holstein-Friesian, and 1394 Limousin bulls. The position of the SNPs in the BovineHD BeadChip genotype panel was based on the UMD 3.1 build of the bovine genome [40]. Excluded were single nucleotide polymorphisms on the X and Y chromosomes, SNPs without a reported chromosome or position, SNPs with a call rate of less than 95%, and SNPs whose genotypes were inconsistent with Mendelian inheritance in more than 2% of the parent-progeny pairs based on a population of 2291 parent-progeny pairs [41]; after edits 713,162 SNPs remained.

### CNV calling software

PennCNV [10] and QuantiSNP [11] are CNV calling algorithms used to call CNVs from raw SNP data. Both algorithms use hidden Markov models to detect CNVs based on the LRR and BAF of SNPs. The LRR of a SNP is the log of the observed probe hybridization intensity divided by the expected probe hybridization intensity. The expected probe hybridization intensity is the intensity that was observed in a reference sample; it is a measure of the fluorescence intensity produced by hybridization of a probe to a SNP array. The BAF is the proportion of B alleles at a SNP. PennCNV requires a CNV to contain a minimum of three consecutive SNPs. Therefore the minimum number of SNPs for a CNV called by PennCNV or QuantiSNP was set to three; this applied to CNVs called from both the high-density and the medium-density genotypes separately. No upper threshold for the number of SNPs per CNV was specified. Diskin et al. [42] reported that the median LRR value of a 1 Mb region of the genome correlates with the guanine-cytosine (GC) content of DNA in that region. The GC adjustment was applied to account for the correlation between the LRR value of SNPs and the GC content of the genome 500 kb flanking either side of the SNP. The GC content of the genome was calculated from the UMD_3.1.1 / bosTau8 genome, complied as of June 2014.

### Comparison of CNVs from high-density and medium-density SNP genotypes

A medium-density SNP genotype panel was created for each animal using the edited high-density SNP genotype panel. The medium-density SNP genotype panel contained SNPs that were common between the edited high-density SNP genotype panel and the commercially available BovineSNP50 beadchip (Illumina Inc. San Diego, CA). The medium-density genotype panel used in the present study contained 45,677 SNPs. Copy number variants were called from the high-density genotypes of each animal in the population using both PennCNV and QuantiSNP; CNVs from the medium density panel were called using just PennCNV. The CNVs called from both genotypes panels by PennCNV were compared for each animal. When the genomic position of a CNV called from the high-density genotypes overlapped with the genomic position of a CNV called from the medium-density genotypes in the same animal, the CNVs were said to overlap. The overlapping region was defined as the genomic region that was common to the CNV called from the high-density genotype and the CNV called from the medium-density genotype.

### Copy number variant imputation

Beagle V4.0 [43] and FImpute [44] are two commonly used imputation software suites; in the present study, these software suites were used to impute CNVs from flanking SNP haplotypes. Beagle uses a hidden Markov model approach to impute missing genotype data in individuals based on the haplotype structure in a reference population which contains both the called CNVs and flanking SNPs. FImpute uses a sliding window approach to identify haplotypes that are shared between individuals in the population. Imputation was carried out separately on the same set of CNVs using both FImpute and Beagle; both software suites were run with default settings with an optional parameter to include pedigree information. Within each of the three breeds, the oldest 80% of animals were used as the reference population with the remaining 20% of animals used as the validation population. The same reference and validation populations were used for the imputation with both Beagle and FImpute.

### Copy number variant imputation from SNP genotype data

The dataset of CNVs used for imputation was the set of CNVs which were called by both PennCNV and QuantiSNP using the high density genotypes. A CNV was considered to be called by both PennCNV and QuantiSNP when the CNV was called in the same animal by both algorithms; a difference of one SNP in the end point demarcation of CNVs between PennCNV and QuantiSNP was allowed [11, 22].

A set of CNVs was selected for imputation within each of three breeds separately. These CNVs were selected based on population frequency; CNVs which were present in at least 30 animals in the breed were selected for imputation, leading to 40 CNVs being selected in Limousin, 36 in Charolais, and 24 in Holstein-Friesian. The reason for selecting CNVs which were present in at least 30 animals within breed was to avoid small sample bias when comparing the imputed copy number of the CNV to the called copy number of the CNV.

For imputation, the selected CNVs were recoded as variants; the actual position chosen for the variant was the midpoint of the CNV. For imputation using Beagle, each CNV was represented as a tri-allelic variant where each allele could be a deletion, a duplication, or normal (i.e. the absence of a deletion or duplication). A double deletion was represented as a homozygous deletion, a single deletion was a heterozygous deletion normal, a normal variant was homozygous normal, a single duplication was a heterozygous duplication normal, and a double duplication was a homozygous duplication. Unlike Beagle which is capable of imputing multi-allelic markers, FImpute can only use bi-allelic markers for imputation; therefore, to impute CNVs which are tri-allelic using FImpute, deletions and duplications were imputed separately. Imputation was performed separately with 10, 25, 50, 100, 250, and 500 SNPs flanking each side of the midpoint of the CNV for both FImpute and Beagle. The SNPs used for imputation flanked the midpoint of the CNV; as such some of the selected SNPs were within the bounds of the CNV and the remaining SNPs flanked the end points of the CNV.

### Statistical analysis for imputation

A CNV was deemed to be correctly imputed when the copy number of the imputed CNV matched the copy number of the called CNV. The imputation accuracy was calculated per CNV as the number of animals in the validation population with a correctly imputed CNV, divided by the total number of animals in the validation population; this calculation was performed within each breed separately. The imputation accuracy was calculated separately for each copy number as called by PennCNV and QuantiSNP. The adjusted Rand index [45] was used to assess the agreement between the called copy number of the CNVs and the imputed copy number of the CNVs. The adjusted Rand index is a method for comparing the agreement between clustering solutions that adjusts for chance agreement [45]. The adjusted Rand index can have values between − 1 and 1; a value of 1 corresponds to perfect agreement, a value of 0 is the expected value for agreement between random clusters, and negative values represent less agreement between groups than would have been expected by chance [46].

To identify factors which may have impacted the accuracy of imputation, an ANOVA, in conjunction with a Tukey’s range test [47], was used to compare the mean imputation accuracy between groups defined by: 1) the number of flanking SNPs, 2) the different copy numbers of the CNVs, and 3) the three different breeds. The Pearson correlation coefficient was used to calculate the correlation between the accuracy of imputation and the population frequency of the CNV, as well as between the accuracy of imputation and the genomic length of the CNV. For each correlation, Fischer’s r to Z transformation [48] was used to calculate the 95% confidence interval for the correlation coefficient; correlations where the 95% confidence interval included zero, were not considered different from zero. QuantiSNP reports the Bayes factor for each CNV; the Bayes factor is a model comparison metric that reports the preference in the data for one model over another [49]. The Bayes factor is a measure of whether the data supports a CNV being called a ‘true’ CNV in that animal. PennCNV does not report the mean Bayes factor of a CNV. An ANOVA analysis was used to determine if there was a difference in the Bayes factor between CNVs where the called and imputed copy number matched, and CNVs where the called and imputed copy number did not match.

## Supplementary information

**Additional file 1: Fig. S1.** Scatter plot of the percentage imputation accuracy against the percentage population frequency of each CNV. A CNV was deemed to be correctly imputed when the called copy number matched the imputed copy number. The red circles represent double deletions (*n* = 9 in Charolais, 15 in Limousin and Holstein-Friesian), green triangles represent single deletions (*n* = 34 in Charolais, *n* = 38 in Limousin, and 22 in Holstein-Friesian), blue squares represent normal state (*n* = 36 in Charolais, 40 in Limousin, and 24 in Holstein-Friesian), double duplications are represented by a purple cross (*n* = 2 in Charolais, *n* = 1 in Limousin and Holstein-Friesian).

**Additional file 2: Fig. S2.** Scatter plot of percentage imputation accuracy against genomic length of CNVs. A CNV was deemed to be correctly imputed when the called copy number matched the imputed copy number. The red circles represent double deletions (*n* = 9 in Charolais, 15 in Limousin and Holstein-Friesian), green triangles represent single deletions (*n* = 34 in Charolais, *n* = 38 in Limousin, and 22 in Holstein-Friesian), blue squares represent normal state (*n* = 36 in Charolais, 40 in Limousin, and 24 in Holstein-Friesian), double duplications are represented by a purple cross (*n* = 2 in Charolais, *n* = 1 in Limousin and Holstein-Friesian).

**Additional file 3: Table S1.** The first quartile, median, and third quartile of the accuracy of imputation of CNVs grouped by called copy number and breed. The number of CNVs in each group is also given. Summary statistics for duplications (*n* = 4) were not included because for each duplication the imputed copy number did not match the called copy number.

**Additional file 4: Table S2.** The location and population frequency of CNVs with an accuracy of at least 85% within at least one of the three breeds. The population frequency is the number of times the CNV was present in the total population, i.e. the reference and validation population. The accuracy, given as a percentage, is the number of times the CNV was accurately imputed divided by the number of times that CNV was called in the validation population. Where an accuracy of NA is reported, imputation was not undertaken for that CNV in that breed.

## Data Availability

The datasets used and/or analysed during the current study are available from the corresponding author on reasonable request.

## Abbreviations

ANOVA: analysis of variance
BAF: B allele frequency
CNV: copy number variant
GC: guanine-cytosine
indel: insertion deletion
kb: kilobase
LRR: log R ratio
MAF: minor allele frequency
SNP: single nucleotide polymorphism
