# Supplementary figures and images for: Concordance rate between copy number variants detected using either high- or medium-density single nucleotide polymorphism genotype panels and the potential of imputing copy number variants from flanking high density single nucleotide polymorphism haplotypes in cattle
Source: BMC Genomics. 2020 Mar 4;21:205. doi: 10.1186/s12864-020-6627-8 (PMC7057620; doi:10.1186/s12864-020-6627-8)

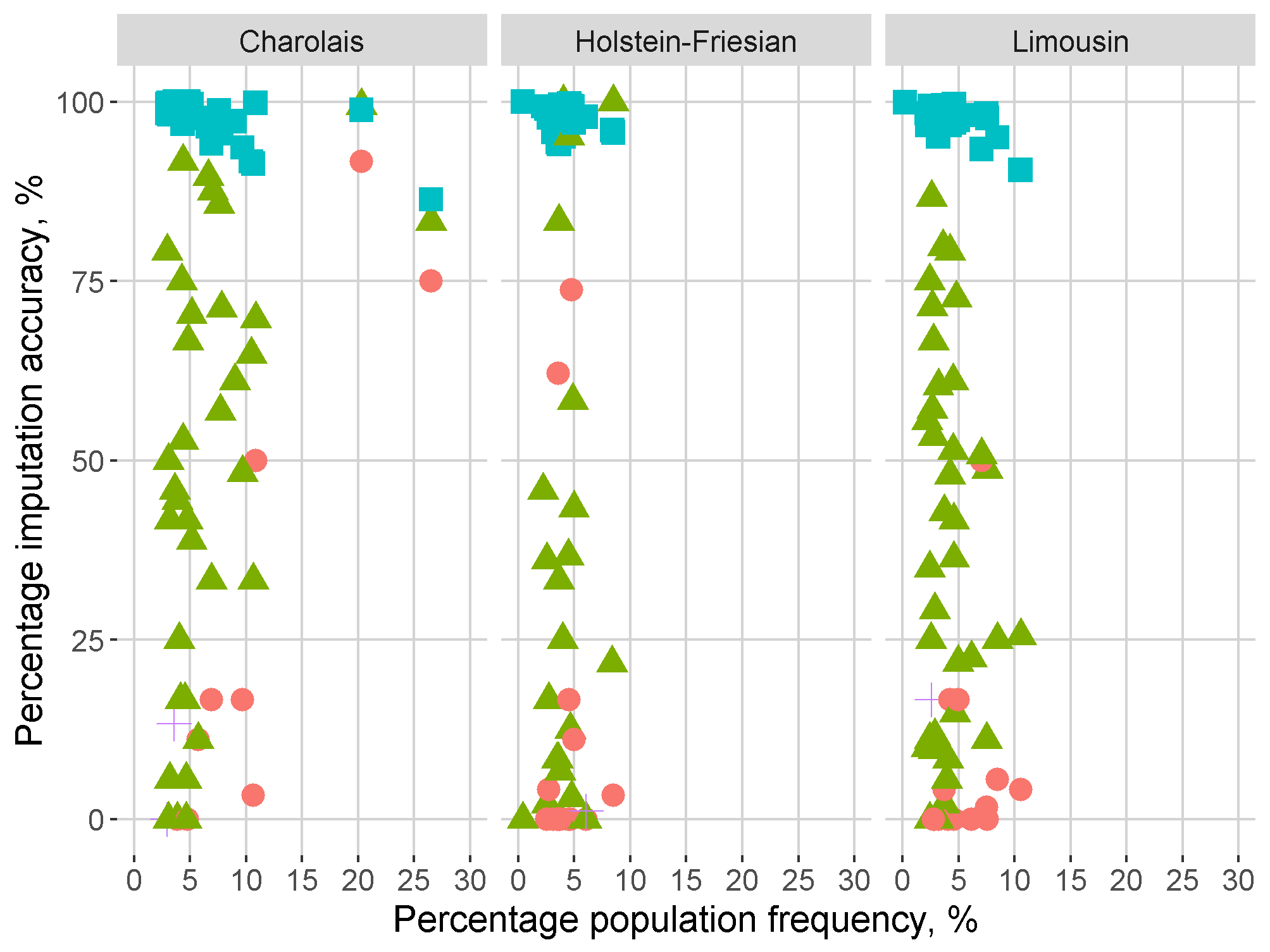

Supplement: Supplementary file 1 — Additional file 1: Fig. S1. Scatter plot of the percentage imputation accuracy against the percentage population frequency of each CNV. A CNV was deemed to be correctly imputed when the called copy number matched the imputed copy number. The red circles represent double deletions (n = 9 in Charolais, 15 in Limousin and Holstein-Friesian), green triangles represent single deletions (n = 34 in Charolais, n = 38 in Limousin, and 22 in Holstein-Friesian), blue squares represent normal state (n = 36 in Charolais, 40 in Limousin, and 24 in Holstein-Friesian), double duplications are represented by a purple cross (n = 2 in Charolais, n = 1 in Limousin and Holstein-Friesian). [file 12864_2020_6627_MOESM1_ESM.tiff]

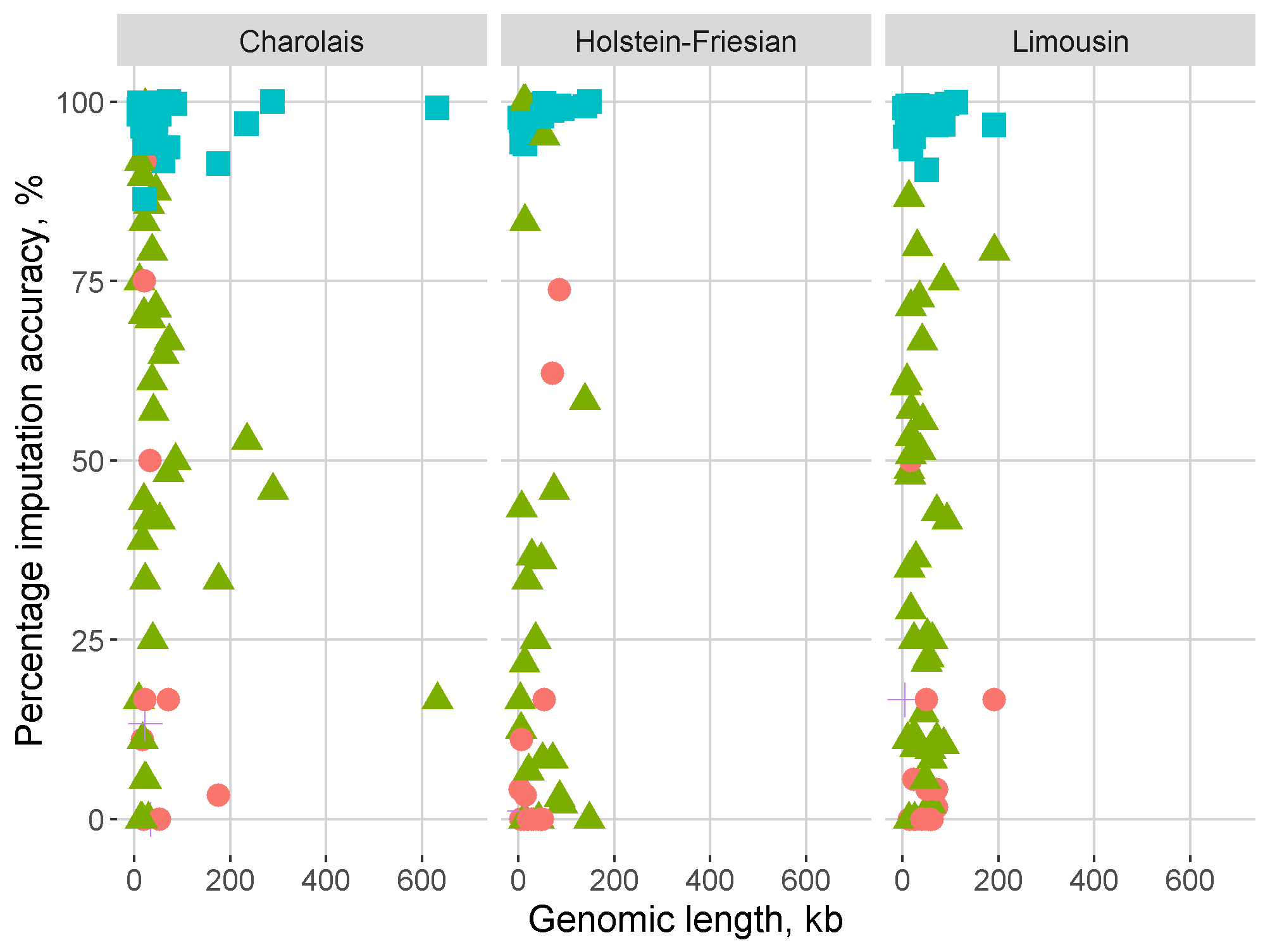

Supplement: Supplementary file 2 — Additional file 2: Fig. S2. Scatter plot of percentage imputation accuracy against genomic length of CNVs. A CNV was deemed to be correctly imputed when the called copy number matched the imputed copy number. The red circles represent double deletions (n = 9 in Charolais, 15 in Limousin and Holstein-Friesian), green triangles represent single deletions (n = 34 in Charolais, n = 38 in Limousin, and 22 in Holstein-Friesian), blue squares represent normal state (n = 36 in Charolais, 40 in Limousin, and 24 in Holstein-Friesian), double duplications are represented by a purple cross (n = 2 in Charolais, n = 1 in Limousin and Holstein-Friesian). [file 12864_2020_6627_MOESM2_ESM.tiff]
