# Supplementary material for: Concordance rate between copy number variants detected using either high- or medium-density single nucleotide polymorphism genotype panels and the potential of imputing copy number variants from flanking high density single nucleotide polymorphism haplotypes in cattle
Source: BMC Genomics. 2020 Mar 4;21:205. doi: 10.1186/s12864-020-6627-8 (PMC7057620; doi:10.1186/s12864-020-6627-8)
Supplement: Supplementary file 3 — Additional file 3: Table S1. The first quartile, median, and third quartile of the accuracy of imputation of CNVs grouped by called copy number and breed. The number of CNVs in each group is also given. Summary statistics for duplications (n = 4) were not included because for each duplication the imputed copy number did not match the called copy number. [file 12864_2020_6627_MOESM3_ESM.docx]

**Table S1**: The first quartile, median, and third quartile of the accuracy of imputation of CNVs grouped by called copy number and breed. The number of CNVs in each group is also given. Summary statistics for duplications (n=4) were not included because for each duplication the imputed copy number did not match the called copy number.

|  | Breed | First Quartile | Median | Third Quartile | Number of CNVs |
| --- | --- | --- | --- | --- | --- |
| Double deletions | Charolais | 0.033 | 0.167 | 0.500 | 9 |
|  | Limousin | 0.000 | 0.000 | 0.049 | 15 |
|  | Holstein-Friesian | 0.000 | 0.000 | 0.076 | 15 |
|  |  |  |  |  |  |
| Single deletions | Charolais | 0.271 | 0.492 | 0.710 | 34 |
|  | Limousin | 0.113 | 0.357 | 0.568 | 38 |
|  | Holstein-Friesian | 0.071 | 0.234 | 0.452 | 22 |
|  |  |  |  |  |  |
| Normal | Charolais | 0.972 | 0.986 | 0.991 | 36 |
|  | Limousin | 0.971 | 0.980 | 0.990 | 40 |
|  | Holstein-Friesian | 0.967 | 0.978 | 0.993 | 24 |
