# Supplementary material for: Concordance rate between copy number variants detected using either high- or medium-density single nucleotide polymorphism genotype panels and the potential of imputing copy number variants from flanking high density single nucleotide polymorphism haplotypes in cattle
Source: BMC Genomics. 2020 Mar 4;21:205. doi: 10.1186/s12864-020-6627-8 (PMC7057620; doi:10.1186/s12864-020-6627-8)
Supplement: Supplementary file 4 — Additional file 4: Table S2. The location and population frequency of CNVs with an accuracy of at least 85% within at least one of the three breeds. The population frequency is the number of times the CNV was present in the total population, i.e. the reference and validation population. The accuracy, given as a percentage, is the number of times the CNV was accurately imputed divided by the number of times that CNV was called in the validation population. Where an accuracy of NA is reported, imputation was not undertaken for that CNV in that breed. [file 12864_2020_6627_MOESM4_ESM.docx]

**Table S2.** The location and population frequency of CNVs with an accuracy of at least 85% within at least one of the three breeds. The population frequency is the number of times the CNV was present in the total population, i.e. the reference and validation population. The accuracy, given as a percentage, is the number of times the CNV was accurately imputed divided by the number of times that CNV was called in the validation population. Where an accuracy of NA is reported, imputation was not undertaken for that CNV in that breed.

| CNV genomic location | Limousin | | Charolais | | Holstein-Friesian | |
| --- | --- | --- | --- | --- | --- | --- |
|  | Population frequency | Accuracy, % | Population frequency | Accuracy, % | Population frequency | Accuracy, % |
| 3:41860820-41883352 | 3 | NA | 206 | 99.4 | 1 | NA |
| 5:41517287-41528650 | 0 | NA | 44 | 91.67 | 0 | NA |
| 6:12447020-12464858 | 36 | 57.14 | 67 | 89.58 | 0 | NA |
| 1:91350265-91395660 | 9 | NA | 71 | 87.5 | 1 | NA |
| 7:10216191-10270468 | 0 | NA | 0 | NA | 45 | 95.24 |
| 15:81030080-81042709 | 35 | 86.67 | 4 | NA | 83 | 0 |
|  |  |  |  |  |  |  |
